# Racial IQ Differences among Transracial Adoptees: Fact or Artifact?

**DOI:** 10.3390/jintelligence5010001

**Published:** 2016-12-23

**Authors:** Drew Thomas

**Affiliations:** Department of Physics, Imperial College London, London SW7 2AZ, UK; dmt107@imperial.ac.uk

**Keywords:** race, IQ, transracial adoption, statistical methodology, Flynn effect, attrition

## Abstract

Some academic publications infer from studies of transracial adoptees’ IQs that East Asian adoptees raised in the West by Whites have higher IQs than Western Whites, and that White adoptees raised by Whites have higher IQs than Black adoptees raised by Whites. Those publications suggest that this is because genetic differences give East Asians a higher mean IQ than Whites, and Whites a higher mean IQ than Blacks. This paper proposes a parsimonious alternative explanation: the apparent IQ advantage of East Asian adoptees is an artifact caused by ignoring the Flynn effect and adoption’s beneficial effect on IQ, and most of the IQ disadvantage of Black adoptees disappears when one allows for attrition in the Minnesota Transracial Adoption Study, and acknowledges the results of other studies. Diagnosing these artifacts suggests a nil hypothesis: East Asian, White, and Black adoptees raised in the same environment would have similar IQs, hinting at a minimal role for genes in racial IQ differences.

## 1. Introduction

Generally, Black people, White people, and East Asian people have different average IQs, but the specific reasons for this remain controversial. Of various bodies of evidence, one has been nominated as “one of the most powerful” for explaining these racial IQ differences: the IQs of adoptees of different races raised by White adoptive parents [1] (p. 25). Several psychologists have reviewed studies of transracial adoptees’ IQs and used them as evidence for two claims. The first is that East Asian adoptees raised by Whites have higher average IQs than the general White population; that White adoptees raised by Whites in turn have higher average IQs than Black adoptees raised by Whites; and that multiracial adoptees have an expected IQ that is a weighted average of their ancestral groups’ mean IQs (so that adoptees born to a White genetic parent and a Black genetic parent, for example, have an average IQ midway between those of Whites and Blacks). This would suggest that racial IQ differences persist even among children raised in nurturing adoptive homes, intimating that differences in home environments cannot explain racial IQ differences. This leads into the second claim: that most of the racial IQ differences between East Asians, Whites, and Blacks are genetic [1,2,3]. In this paper, I call the conjunction of these two claims the *hereditarian* hypothesis or model, after Rushton and Jensen [3] (pp. 239–240, 259).

This paper reanalyzes the adoption studies cited to support these claims, and puts forth an alternative explanation: most of the average racial IQ differences in those studies are spurious, arising from methodological issues and disregard of contrary results. When all of the cited data are considered with these issues in mind, they are not compelling evidence for large and consistent IQ differences between East Asian, White, and Black adoptees raised by White parents. This paper then introduces further adoption data which have yet to be considered in the race and IQ debate. The totality of the data turn out to be at least as consonant with a *nil* hypothesis or model: the IQs of adoptees raised by Whites in comparable environments are hardly affected by the adoptees’ race.

## 2. Methodological Issues

The innovation of this paper is that it accounts for four methodological issues which imperil analyses of transracial adoption studies of IQ. Two of the issues are examples of the generic scientific problem of confounded comparisons of groups, and the third is a type of selection bias.

Firstly, if a sample of adoptees of one race or ethnicity scores above another ethnic group’s general-population average, one cannot automatically attribute the above-average score to the adoptees’ ethnicity. The adoptees are *adoptees*, and adoptees are typically raised in unrepresentative environments which tend to be more nurturing and high in socioeconomic status. Unusually wholesome environments could then explain the adoptees’ above-average IQ, rather than the adoptees’ race; race and environment would be confounded. (Indeed, the adoptees themselves are likely to be unrepresentative of their own race, and a sceptic could conceivably attribute the adoptees’ above-average IQ to their very unrepresentativeness, regardless of environment.). The two most obvious ways to control away this confounding are to compare adoptees against only other adoptees, or to adjust the adoptees’ observed IQs downwards to allow for the adoptees’ better environments.

The second methodological issue is the Flynn effect. James R. Flynn and his colleagues have documented steady rises in average IQ in several countries [4,5,6,7,8]. These rises makes IQ test norms progressively more outdated over time, so adoptees who take an IQ test would have exaggerated IQ scores relative to people who took the same test earlier. In particular, all IQ tests must be standardized against a reference population, offen the general population of a given country, and when a group of adoptees takes the IQ test at a later date, the time lag exaggerates the adoptees’ performance relative to the reference population. Had the reference population taken the test at the same time as the adoptees, the reference population would have set a higher benchmark for the adoptees. The same mechanism means it is usually illegitimate to directly compare adoptees’ IQs across studies, because adoptees in different studies are usually tested in different years, and with different tests. Like environment, the year in which a test is taken and the choice of test can be confounded with race. To avoid such confounds, analysts can either subtract out the Flynn effect from each set of results, or make comparisons only of groups which took the same IQ test at approximately the same time.

The third issue, attrition, is less common, affecting only longitudinal studies. Even if a longitudinal study compares adoptees against only other adoptees (eliminating the first confound) who took the same IQ test at similar times (eliminating the second confound), attrition can take place between waves. When researchers lose track of some subjects between waves of a longitudinal study, the pattern of subjects lost to follow-up can vary between subgroups of subjects, degrading the statistical comparability of those subgroups. Taking the specific case of adoptees’ IQs, if a longitudinal study included e.g., White and e.g., Black adoptees, and the White adoptees lost to follow-up were disproportionately lower scorers, whereas the Black adoptees lost to follow-up were not, the White–Black IQ difference among the remaining adoptees would be inflated. Race can correlate with selection for retention in the study. One can adjust for this type of selection bias by making a counterfactual estimate of how the subgroups would have scored had no one been lost to follow-up.

The fourth issue is that published reviews of transracial-adoption IQ studies have not considered all of the studies which were available and germane, and this selective treatment of the data may introduce bias. Consequently, as Rushton and Jensen put it, “[t]o be compelling, [...] researchers must take the totality of available evidence into account” [9] (p. 921). The remainder of this paper should give an idea of whether past writers on transracial adoption and IQ have met this standard.

## 3. A Hereditarian Interpretation

Before contesting the hereditarian model of transracial adoption studies, this paper must first describe it. To that end, this section takes stock of the studies already discussed in the literature, and why they might seem to support the hereditarian hypothesis. Table 1 gives a list in publication-year order, where samples of adoptees with two Black genetic parents each are labelled “Black–Black” and samples of adoptees with one White and one Black genetic parent each are labelled “Black–White”. The 130 East Asian adoptees in the Winick, Meyer, and Harris [10] and Frydman and Lynn [11] studies were all Korean, while the 25 East Asian adoptees in the Clark and Hanisee [12] sample comprised “12 from Vietnam, 8 from Korea, 3 from Cambodia, and 2 from Thailand” (p. 596). The racial designations may not be rigorous but the current paper uses them for argument’s sake.

A hereditarian interpretation of the data in Table 1 begins by “plac[ing] greatest weight on the Minnesota Trans-Racial Adoption Study because it is the largest and best-known of these studies and is the only one that included a longitudinal follow-up” [1] (p. 25). Scarr and Weinberg [13] reported results from the first wave of that study, and Weinberg, Scarr, and Waldman [14] the follow-up results. In both waves the wholly White adoptees had a higher mean IQ than the Black–White adoptees, and the Black–White adoptees had a higher mean IQ than the Black–Black adoptees, in line with the hereditarian model. Moreover, those IQ gaps widened between the study’s two waves, consistent with genes exerting greater power as the adoptees aged.

The other studies of Black adoptees [15,16] do not fit the hereditarian model nearly as well, as the race-IQ correlations they found were all either small or associated higher IQ with greater Black ancestry. However, one can downplay these other studies on the ground that they lacked longitudinal follow-up testing, and by arguing that genetic effects could’ve been masked by their subjects’ lower age (since “as people age, their genes exert ever more influence” [3] (p. 259)).

This aligns with the hereditarian claim that mean IQ decreases as Black ancestry increases. As for the claim that mean IQ rises with greater East Asian ancestry, there is the fact that three studies of East Asians adopted in Western countries each reported higher mean IQs for them than the norm of 100 [3] (pp. 259–260), as can be seen in Table 1. The lower mean IQ for adoptees with more Black ancestry and the higher mean IQ for East Asian adoptees may be read as support for the hereditarian model.

## 4. A Re-Analysis of East Asian Adoptee IQ Data

However, the high IQ of these studies’ East Asian adoptees is misleading because none of the studies included adoptees of other races, so they had no direct control groups. This posed the two confounding problems introduced in Section 2 above: the Flynn effect and the IQ boost from the adoptive environment.

Adjusting for the Flynn effect is conceptually trivial: look up which test an adoptee sample took; work out how much time elapsed between when the test was standardized and when the adoptees took the test; look up the rate of IQ gains for the general population where the adoptees were raised; finally, multiply that rate by the time elapsed between standardization and the adoptees taking the test for an estimate of how much the Flynn effect inflated the adoptees’ average IQ. Subtracting that estimate from the adoptees’ measured IQ then gives a truer estimate of their IQ.

Adjusting for the adoptive IQ boost is harder. Theoretically one could estimate the average IQ boost due to adoption and subtract it from an adoptee sample’s average IQ, but which estimate to use for the average IQ boost from adoption is unclear. The meta-analysis of van IJzendoorn et al. [17] has estimates but there are two reasons not to use them. For one thing, the van IJzendoorn et al. meta-analysis incorporated the East Asian adoptee studies I discuss here, so correcting the East Asian IQ means with the van IJzendoorn et al. estimates would be circular reasoning. Worse, van IJzendoorn et al.’s summary of various studies had inaccuracies. For example, their Table 1 (p. 306) described the mean IQ of Korean adoptees in the Winick et al. study [10] as matching a norm of 100 (specifically, the Cohen’s *d* effect size associated with IQ was “0.00”), although one can calculate from Winick et al. [10] that the tested adoptees’ mean IQ was 106.7.

As a result, I have no general estimate of adoption’s positive effect on IQ, which prevents me from adjusting for the adoptive IQ boost confound. Instead, I adjust for the Flynn effect alone, and then simply ask whether any remaining East Asian IQ advantage is small enough that it could plausibly be attributed to the adoptive IQ boost.

Winick et al. [10] is the oldest of the three East Asian adoptee IQ studies. It surveyed 36 “malnourished”, 38 “moderately nourished”, and 37 “well-nourished” Korean girls adopted as infants by Americans. Upon taking IQ tests between 1971 and 1973, the three groups obtained mean IQs of 102, 106, and 112 respectively. Rushton and Jensen [3] (p. 260) observed that they “exceeded the national average” in IQ.

The adoptees’ IQs were measured in school [10] (p. 1173) with four different group tests: the Lorge-Thorndike Intelligence Test, the Otis-Lennon Mental Ability Test, the Cognitive Abilities Test, and the California Test of Mental Maturity (p. 1175). Unfortunately, Winick et al. did not specify which editions of each test were used, so it is impossible to correct exactly for the Flynn effect. The mental-test guide *Intelligence: Tests and Reviews* [18], up to date through January 1974 (p. xxvi), reported that various editions of these tests were published between 1936 and 1972 (pp. 7, 10, 32, 41). The degree to which the Flynn effect inflated the adoptees’ apparent IQs depends on which children took which edition of each test and when.

In the US the population’s IQ has risen by 0.3 IQ points per year [4,5,6,7]. Hence the Flynn effect might have exaggerated the adoptees’ mean IQs by as many as 11 points: multiplying 0.3 points per year by the 37 years between 1973 and 1936—when the oldest versions of the tests were published—gives an 11.1-point IQ inflation. (Were the 1936 tests standardized some time before being published the effect could be even greater.) Deducting 11 points from each group mean gives an overall mean IQ of 96, illustrating that the Flynn effect could account for all of the apparent IQ advantage in Winick et al. [10]. At the other extreme, the adoptees’ schools might have tested almost all of the adoptees in 1972 with the 1972 edition of the Cognitive Abilities Test, in which case the Flynn effect would have inflated the adoptees’ IQ scores only negligibly.

Given the study’s age, the records of how each child was tested are likely lost, which bars an exact estimate of the Flynn effect affecting this study. Splitting the difference between the two extreme scenarios suggests a Flynn effect of 5–6 points. The studies’ results are therefore consistent with a baseline IQ of 100 for Korean adoptees, exaggerated by a Flynn effect of 6 points and an adoptive IQ boost of 6 points, with the undernourished groups losing 6–10 points through malnourishment. Rushton and Jensen’s contention [3] (p. 260) that the adoptees’ IQ “exceeded the national average” is quite possibly fallacious.

A reviewer has suggested that the “malnourishment might be a genetic effect”, because low-IQ parents “are poor and have less money for food for their children”. Whether this has any effect on my argument depends on the precise comparison one has in mind. If the relevant comparison is between East Asians and Whites with identical environments, including identical levels of nutrition, then whether real-world malnutrition is linked to genes is irrelevant; one wishes to equalize the levels of nutrition between the comparison groups regardless, and may write off the lower IQ of the less nourished groups as the result of an unfair comparison. Alternatively, if one thinks the relevant comparison is between East Asians and Whites born in environments that may differ as long as the differences can be attributed to genes, then correcting for malnutrition is an overcorrection insofar as the malnutrition is genetically driven.

Supposing the latter, *could* genes suffice to explain the lower IQs of the less nourished groups? The causal chain the reviewer highlights runs from parental genes to parental IQ to family income/wealth to child’s malnutrition to child’s IQ. To set an upper bound on the overall strength of this chain, I may take the product of the upper limits of the correlations between each consecutive pair of variables, assuming arguendo that the links between each pair are causal and linear.

The first correlation is the square root of IQ’s heritability among the parental population; a high heritability figure of 80% gives a correlation of 0.89. For the correlation between IQ and income I use 0.23, the highest value from the meta-analysis of Strenze [19] (p. 412); note that this is Strenze’s correlation between individual IQ and individual income, which is presumably higher than that between individual IQ and *family* income. Lacking a convenient meta-analysis or large-scale study quantifying the correlation between family affluence and malnutrition, I will be generous to the reviewer’s argument and suppose the correlation is implausibly high, say 0.8. The final correlation is that between child malnutrition and child IQ, which one may estimate from the Winick et al. [10] results themselves, using height and weight percentiles as a quantitative nutritional index. The children in that study designated “malnourished” or “well-nourished” were “below the 3rd percentile for both height and weight” and “at or above the 25th percentile for both height and weight” respectively (p. 1173), and so (making the approximation that height and weight were normally distributed in the reference population) the two groups were separated by at least 1.88 − 0.67 = 1.21 standard deviations in nutritional level. However, the two groups’ mean IQs differed by only 10 points, or two thirds of a standard deviation, implying a nutrition-IQ correlation of at most (2 ÷ 3) ÷ 1.21 = 0.55. The overall chained correlation is then 0.89 × 0.23 × 0.8 × 0.55 = 0.09. To explain the two-thirds-of-a-standard-deviation discrepancy between the least and most nourished subsamples in the Winick et al. study, therefore, one would have to posit that the difference in parental IQ between the two subsamples was (2 ÷ 3) ÷ 0.09 = 7.4 standard deviations. This is implausible. The assumptions feeding into that estimate are themselves collectively implausible, but they are conservative, so substituting more plausible assumptions would not change the qualitative result: the chained effect of genes on IQ, of IQ on income, of income on nutrition, and of nutrition on IQ is too feeble to explain away the IQ differences between Winick et al.’s groups on the reviewer’s genetic grounds.

This frees me to move on to the most recent of the three East Asian adoptee IQ studies, namely Frydman and Lynn [11], a short report on “19 Korean children who were orphaned or abandoned in Korea in the mid-1970s and subsequently adopted by Belgian families” (p. 1323). In 1983 the children attained a mean IQ of 118.7 on the Wechsler Intelligence Scale for Children (WISC), with a verbal mean (VIQ) of 110.6 and a performance mean (PIQ) of 123.5. Frydman and Lynn recognized that they oughtn’t take these scores at face value, observing that “the French WISC was standardised in 1954, […] and that the mean IQ in Belgium will have increased over the 29-year period between the standardisation and the testing of the Korean children” (p. 1324). Unfortunately, when adjusting for this, they then assumed that IQ had risen in Belgium at the same rate as in the US: “3 IQ points per decade” (p. 1324).

This was almost surely an under-adjustment. Flynn [5] (p. 185) had already published Belgian military results from samples of 18-year-old men, clocking nonverbal test gains at 7–8 points per decade and verbal test gains at 4 points per decade. Mapping the verbal gains to VIQ and the performance gains to PIQ and deducting both from the adoptees’ means reduces the adoptees’ mean VIQ to 99 and their mean PIQ to 100–103. Adjusting properly for the Flynn effect, then, the Frydman and Lynn adoptees scored on par with Belgian children. When one recalls that adoption should have raised the adoptees’ IQs above the general population’s average, this outcome is surprising; it suggests that Frydman and Lynn’s subjects would have scored *below* comparable White adoptees in Belgian homes.

Frydman and Lynn acknowledged that the Korean adoptees “were brought up in middle class families” and that this “would have raised their mean IQ above that of all [sic?] Belgian children” in itself (p. 1324). Estimating that “middle class children obtain a mean IQ of about 105”, Frydman and Lynn interpreted their under-adjusted mean of 108.7 as one that “would suggest a genotypic Korean advantage” (p. 1324), but this latter conclusion was an error. After a more sensible adjustment, the Korean adoptees’ true mean VIQ and PIQ were between 99 and 103, both less than 105. By Frydman and Lynn’s logic this would suggest a genotypic Korean IQ *disadvantage*.

A possible rebuttal to my argument is that I am over-adjusting by using Flynn’s data on Belgian IQ gains. Flynn’s data cover only the period of 1958 to 1967, whereas Frydman and Lynn’s adoptees could have been born no earlier than 1969. Possibly Belgian IQ gains were slower while Frydman and Lynn’s subjects were growing up, leading me to adjust the adoptees’ averages too much.

Without additional Belgian data it is impossible to decisively refute this rebuttal, but records of French IQ gains, a proxy for Belgian gains, weigh against it. Military samples of French men aged 18 to 22 revealed gains of a point a year on Raven’s Progressive Matrices and 0.4 points per year for verbal and mathematical tests between 1949 and 1974, gains at least as fast as those in the Belgium data [5] (p. 185). Flieller et al. [20] documents a gain of 1.6 standard deviations on Gille’s Mosaic test for French 8-year-olds between 1944 and 1984, the equivalent of 0.6 IQ points per year. Deducting 29 × 0.6 = 17.4 points from the adoptees’ full-scale IQ mean of 118.7 brings it down to 101.3, neither practically nor statistically different from 100, and again less than 105. Flynn [5] (pp. 184–185) also records French gains on the WISC between 1955 and 1979 among 6–15-year-olds, with PIQ rising by 0.6 points a year and VIQ by 0.1 points a year. The minuscule VIQ increase is, however, “unique” and Flynn categorizes the French WISC data as “speculative”.

The overall pattern of French results fits my estimates of Belgian IQ gains. All in all, it is very likely that the Korean adoptees in Frydman and Lynn [11] would have scored little better than comparable White adoptees. This cuts against Rushton and Jensen’s assertion [3] (p. 260) that “the Korean children still had a statistically significant 10-point advantage in mean IQ over indigenous Belgian children”. The allegation that “[n]either the social class of the adopting parents nor the number of years the child spent in the adopted family had any effect on the child’s IQ” (p. 260) was also an overreach. For one thing, Frydman and Lynn merely presented bivariate correlations between IQ and whether the parents had a university degree, and between IQ and the number of years the adoptee spent with the adoptive family, and it is impossible to make a conclusive causal statement such as Rushton and Jensen’s from those correlations alone. For another, whether or not parents have a university degree is a blunt measure of social class. For a third, the study’s sample size was only 19, so the study would have had mediocre power to detect a correlation.

This leaves the study of Clark and Hanisee [12], in which 25 adoptees raised in the US had an average IQ-equivalent of 120 on the Peabody Picture Vocabulary Test (PPVT). *Intelligence: Tests and Reviews* [18] (p. 930 or §7:417) shows that the PPVT dates from 1959, so the test norms were presumably 20–25 years old when the 25 adoptees were tested. I deduct 6–8 points accordingly for a Flynn-adjusted mean of 112–114. As this is far above the norm of 100, the Flynn effect does not wholly explain these adoptees’ elevated IQ. Possibly the adoptees’ PPVT scores were inflated further by the PPVT’s unrepresentative standardization “on 4012 white children and youth in Nashville, Tennessee” [18] (p. 752 or §6:530), but it is not obvious why that would explain all of the adoptees’ extra 12–14 IQ points.

One reviewer has intimated that I overcorrected for the Flynn effect in Clark and Hanisee’s sample because the PPVT measured the “narrow ability” of verbal ability, while my 3-point-per-decade correction comes from broad IQ batteries. My counter-counterargument: verbal ability is broad, not narrow [21], and even vocabulary tests can show a hefty Flynn effect. I am unaware of Flynn-effect data for the PPVT, but in the US National Longitudinal Survey of Youth, standardized scores on the PPVT-R increased by 0.41 standard deviations in 14 years, equivalent to 4.4 points per decade [22].

Clark and Hanisee hazarded that the elevated IQ might “be the result of adoptive home environment. […] Only 1 child was adopted into a family with an annual income of less than $15,000, whereas 11 were placed in families earning more than $25,000 per year (1978 figures). Both parents typically had college degrees” (p. 597). This would be consistent with a French study which found that abandoned children adopted into upper-middle-class families had IQs 11–16 points higher than half-siblings and full siblings who remained with their biological mother [23]. Of course, the applicability of those results to East Asian children adopted in the US is not guaranteed, and other explanations of the Clark and Hanisee results exist.

For instance, one could take the observed mean IQ of 120 and deduct only a few points each for the Flynn effect and the boost from adoption, attributing the remaining excess to a genetic advantage. One could even argue that were it not for the harsh environments the adoptees suffered early in life, their IQs would be even higher, suggesting an even greater genetic advantage. Most of the sample’s Vietnamese adoptees “were evacuated from Vietnam during the last stages of United States military involvement in 1975” (p. 597), and all of the sample had lived in an orphanage, foster home, or hospital. Taking this route, Rushton and Jensen [3] wrote that “half of the babies had required hospitalization for malnutrition” (p. 260), implying that the adoptees scored well in spite of needing hospital care. In this vein, I note that Clark and Hanisee [12] actually report a correlation of +0.3 between hospitalization and PPVT score, and indeed a correlation of +0.7 between hospitalization and scores on another test, the Vineland Social Maturity Scale (p. 598).

This completes my reexamination of these three East Asian adoptee studies. After allowing for the Flynn effect, the Clark and Hanisee sample (*N* = 25) had a mean vocabulary-as-IQ of 112–114, the Frydman and Lynn sample (*N* = 19) a mean VIQ of 99 and a mean PIQ of 100–103, and the Winick et al. sample (*N* = 111) a mean IQ of 96–107. These averages do not allow for the adoptive IQ boost, about which one may only speculate. In my judgement, allowing for the adoptive IQ boost would almost certainly bring the Frydman and Lynn averages below 100, would more likely than not bring the Winick et al. average below 100, and would shift the Clark and Hanisee average to approximately 105. Bearing the studies’ sample sizes in mind, these averages imply a slight IQ *dis*advantage for East Asian transracial adoptees, although the evidence leans only modestly in this direction. Conservatively, I infer that East Asian transracial adoptees would score about as well as White adoptees raised in the same homes.

## 5. A Re-Analysis of Black Adoptee IQ Data

Unlike the studies of East Asian adoptees, all of the Black adoptee studies include multiple groups with differing racial admixture, tested at about the same time on similar tests. At least in theory, this allows direct comparison of the groups within each study; since the groups all benefit from adoption’s effect on IQ, and their IQs are inflated to similar degrees by the Flynn effect, there is less risk of these effects biasing the results in favour of one racial group over another. However, the biggest of the Black adoptee studies has other complications to untangle.

That biggest study is the Minnesota Transracial Adoption Study (MTRAS). According to Rushton and Jensen, it is “also the only transracial adoption study [of IQ] that includes a longitudinal follow-up” [3] (p. 256). For the study Sandra Scarr and colleagues located White Minnesotans who had adopted non-White children, and recorded the IQs of the adopters and their children (including the non-adopted White children). Scarr et al. measured the children’s IQs in two waves, one when the children had a mean age of 7 and another when they had a mean age of 17. At both times the White adoptees scored higher than the Black–Black adoptees, and the Black–White adoptees scored between the White and the Black–Black adoptees [13,14].

Not only that, but the measured interracial IQ differences grew between the two waves. Scarr and Weinberg [13] reported differences in the first wave of 2.5 points between the White and Black–White (BW) adoptees, and 14.7 points between the White and fully Black adoptees; Weinberg et al. [14] reported final differences of 7.1 points and 16.2 points respectively. Rushton and Jensen [3] (p. 259) implied that this widening was a genetic effect: “although the shared-family environmental component of true-score IQ variance can be quite large at age 7, by late adolescence it is the smallest component. After that age, genetic and within-family (nonshared) environmental effects account for the largest components”. To convince the reader, they pointed to their Figure 3, a plot estimating the proportions of IQ variation “attributable to genetic and environmental (shared and nonshared) effects” with respect to age (p. 252). However, as Richard Nisbett realized, that diagram indicates that “a greater genetic contribution to IQ occurs only after the age of 20” [24] (p. 308), because it shows virtually constant heritability from age 6 to age 20. Rushton and Jensen contradicted their own cited graph.

But perhaps the widening interracial differences in the MTRAS were genetically driven despite Rushton and Jensen’s error? Probably not, because attrition can explain the apparent widening. A total of 25 White adoptees were in the study when it began, nine of whom were lost at follow-up. The lost adoptees had relatively low IQs, so the remaining White adoptees were unrepresentatively high in IQ, as Mackintosh observed [25]. One can prove this by comparing the original IQs of the full sample and the subgroup who were measured at both ages 7 and 17; the latter subgroup had an initial mean IQ of 117.6 (with a minimum IQ of 92) but the full sample had an initial mean of 111.5 (minimum 62). Because initial and final IQs had a correlation of 0.63 among the White group, the elite subgroup would likely have had their final mean IQ inflated by about 0.63 × (117.6 − 111.5) = 3.8 points. Meanwhile, the BW and Black–Black adoptees lost to follow-up hardly differed in IQ from the remaining adoptees, so attrition inflated those groups’ mean IQs by about only 0.2 and −0.7 points respectively.

Adjusting the final mean IQs accordingly (Table 2) implies smaller racial differences of 3.5 points (White vs. BW adoptees) and 11.7 points (White vs. Black–Black adoptees) in the study’s final wave. The former is only 1 point wider than the corresponding initial difference, and the latter is 3 points narrower. Hence, allowing for attrition, the IQ differences between the White and the Black adoptees were no larger at age 17 than at age 7, a sign that the apparent enlarging was an artifact and not a genetic effect.

With the widening explained, the only racial IQ differences left to comment on are those present at initial testing. The scant initial gap of 2.5 ± 3.5 points between the fully White and BW adoptees is small enough to be simple statistical noise. Only the IQ of the Black–Black adoptees, who scored 12.2 ± 2.8 points below the BW adoptees, calls for a specific explanation. Differences in home environment are one possibility. On every reported environmental variable, the Black–Black adoptees were worse off than both the BW and fully White adoptees, which I quantify by comparing the former against the BW adoptees, measuring the environmental differences in BW SDs. I use the BW adoptees as a comparison group here because Scarr and Weinberg [13] present more data for BW adoptees than White adoptees. The Black–Black adoptees were older when adopted (by 2.1 SDs, or two years); had spent less time in their adoptive home (by 1.1 SDs); had more (by 0.4 SDs) and lower-quality (by 0.8 SDs) adoptive placements; and had adoptive parents with less education and lower mean IQ (by 0.2–0.3 SDs). Additionally, 97% of the BW adoptees had White mothers while the Black–Black adoptees all had Black mothers, with whatever prenatal environmental differences that entailed.

Proponents of the hereditarian model have found the notion of confounding with home environment controversial. For instance, Lee [26] (p. 253) found confounding “very doubtful” because “[t]here exists no independent evidence that variables such as age at adoption exert effects on IQ lasting until late adolescence”, citing the van IJzendoorn et al. meta-analysis [17]. However, as mentioned above, that meta-analysis erroneously summarized its studies, and so its analyses (being based on mis-estimated summary statistics) are untrustworthy. Even ignoring this problem, the meta-analysis claimed low power to detect an adoptive-age effect on IQ; the IQ differences associated with higher adoptive age had wide confidence intervals and a lot of heterogeneity (p. 311). Lee added that “the proportion of IQ variance associated with these pre-adoption variables declined over the course of the MTAS from .32 to .13”. This is true, but I repeat that the only racial IQ differences in the MTRAS needing a special explanation are those measured at age 7, when the pre-adoptive variables had more explanatory power. Lee also made the reasonable if tentative argument that race and IQ themselves might “affect pre-adoption experience”, in which case adjusting for pre-adoptive variables would be “perhaps overly generous towards an environmental hypothesis”. He was correct, but this simply means the MTRAS results are ambiguous; making the adjustment may skew the results in favour of a non-hereditarian hypothesis, but not making the adjustment may skew the results in favour of a hereditarian hypothesis. A decisive, objective, and complete interpretation of the results is not possible.

Malloy [27] presented results from the MTRAS, writing that “no simple or plausible environmental theories […] explain these kinds of findings”, on the grounds that “[s]tudies do not support a large role for peer effects on developed intelligence” and that van IJzendoorn et al.’s meta-analysis “found that neither age at adoption or even coming from an abusive or neglectful environment had an effect on the developed IQ scores of adopted children” (p. 1088). As I do not invoke peer effects on IQ I need not comment on those, and I have already commented on the meta-analysis. I will add that the meta-analysis had poor power to detect the effect of abuse on IQ, which may explain why the abuse-associated deficit found (*d* = 0.22) was statistically insignificant.

Lynn [2] (p. 24) preempted one of Lee’s comments by noting that “what appears to be an age-of-adoption effect may be only a race-differences effect” because correlations between adoptive age and IQ, and between time spent in the adoptive home and IQ, “are confounded with race differences”. Again, this is possible, but simply means the study’s results are ambiguous. (Below I also adduce evidence that adoptive age correlates negatively with IQ among East Asian transracial adoptees, where Lynn’s proposed confounding is excluded.) Lynn makes additional arguments using results for the adoptees at age 17, but the age 7 results are again the pertinent ones.

Rushton and Jensen [3] zeroed in on one particular environmental variable: age at adoption. They referred to Jensen’s 1998 book *The g factor*, which cited Fisch et al. [28], a study supposedly “showing that age of adoption does not influence children’s IQ scores after age 7” [3] (p. 259). However, Nathan Brody [29] (p. 403) noticed that this is a “somewhat tendentious interpretation” of Fisch et al.’s work. Briefly, Fisch et al. compared the IQs of 7-year-olds adopted by their first birthday and 7-year-olds who had been adopted later, discovering a statistically insignificant 4.4-point difference. However, it is unsurprising that this difference was statistically insignificant because “the small sample of [seventeen] adoptees older than 1 renders the power of the statistical test of the difference weak” [29] (p. 402). Rushton and Jensen’s inference that “age of adoption does not influence children’s IQ scores after age 7” stands a good chance of having been a type II error.

The next sentence of Rushton and Jensen’s review was similarly tendentious: “Studies of severely malnourished, late-adopted, East Asian children (see below) provide substantial evidence that age of adoption does not adversely influence IQ in transracial adoptions” [3] (p. 259). The East Asian adoptee studies they referred to are the three I discuss above, yet the adoptees in those studies were *not* “late-adopted” relative to the Black adoptees in the MTRAS, who were adopted at 18 months on average [13] (p. 730). The Winick et al. [10] (p. 1175) adoptees had a mean age at adoption of 18 months and the Frydman and Lynn [11] (p. 1323) adoptees had a mean age at adoption of 19 months. Clark and Hanisee’s paper [12] does not record an average adoptive age, but its adoptees also don’t seem to have been “late-adopted”, as the investigators set an upper adoptive age limit of three years (p. 596), and 10 of its 25 adoptees “were relinquished at birth to adoption agencies” (p. 598). Rushton and Jensen also omitted mention of the negative correlations between adoptive age and IQ documented in Frydman and Lynn [11] and Clark and Hanisee [12]. Winick et al. [10], which paid less attention to adoptive age, does not record an age-IQ correlation, but the follow-up study Lien et al. [30] found a statistically significant negative relationship between academic achievement and age of arrival in the US for Korean adoptees.

There are no features of the Lien et al. study which explain Rushton and Jensen’s omission of it. Lien et al. [30] is a study of Korean adoptees raised in the US with extremely similar design to that of Winick et al., the key difference being that the Lien et al. adoptees were at least two years old when adopted while the Winick et al. adoptees were adopted by age 3. Comparing mean IQs across the studies shows that this adoptive age difference was associated with a 5–7 point IQ deficit for Lien et al.’s later adoptees, regardless of nutritional status.

All in all, confounding of adoptee race with environmental variables is a threat to the MTRAS results. Still another factor complicating the interpretation of the MTRAS results is a hard-to-predict Flynn effect, which seems to be caused by the use of different IQ tests for adoptees of different ages [13,14] and the different age distributions of the White and the Black adoptees [13] (p. 730). Loehlin [31] (p. 185) presented mean IQs for the study’s groups, “adjusted for norm shifts over time”, but his tabulation of the data is too meagre to permit detailed analysis. The original data, which I analyze here, may be skewed by this Flynn effect. Correcting for it could conceivably eliminate the attrition effect while restoring the widening of racial IQ gaps over time, but there is little a priori reason to expect that.

I have had to dwell on the MTRAS at length, but there are two more oft-cited Black adoptee studies. One is Tizard [15], a one-page report spun off from a language-acquisition study. In that study 64 4½-year-olds took the Wechsler Pre-school and Primary Scale of Intelligence (WPPSI) IQ test, of whom 24 “had been adopted into white families at a mean age of 3.1 yr” (p. 316). A total of 17 adoptees were White and had a mean IQ of 113.0, and seven were BW and had a mean IQ of 119.9. The superior IQ of the adoptees with more Black ancestry reverses the main result of Scarr and Weinberg. In Scarr and Weinberg [13], the BW adoptees lagged the White adoptees by 2.5 ± 3.5 points, while in Tizard [15], the BW adoptees outscored the White adoptees by 6.9 ± 6.6 points.

The other study is Moore’s [16], which assessed 23 Black–Black and BW adoptees, raised in White families, on the WISC. Like Tizard [15] and unlike Scarr and Weinberg [13], the adoptees with more Black ancestry had higher IQs: nine fully Black adoptees had a mean IQ of 118.0 and 14 BW adoptees had a mean IQ of 116.5. The resulting IQ difference is 1.5 ± 4.1 points, where the standard error is approximate because the standard deviations involved are pooled estimates.

Taking an inverse-variance-weighted average of results from Scarr and Weinberg and Tizard, BW adoptees lagged White adoptees by 0.4 ± 3.1 IQ points. Taking an inverse variance-weighted average of results from Scarr and Weinberg and Moore, fully Black adoptees lagged BW adoptees by 7.8 ± 2.3 points, though this estimate assumes a homogeneity of results that doesn’t exist. Taken at face value these results suggest that higher Black ancestry is associated with lower IQ among Black adoptees, but not when comparing BW to White adoptees. If one forces these two conflicting results together by taking a weighted average of the two weighted averages, they suggest an IQ drop of about 5 points associated with having an additional Black biological parent, but statistical heterogeneity renders this result suspicious. Another reason for suspicion comes from Moore’s work, which also studied 23 Black adoptees raised in *Black* families. Those adoptees had a mean IQ 13.5 ± 3.1 points below the mean of the Black adoptees raised in White families, evidence for the importance of adoptees’ home environment rather than adoptees’ ancestry.

A hereditarian might invoke heterosis (hybrid vigour) as an explanation for the heterogeneity—perhaps Black ancestry lowers IQ on average, with this effect cancelled out in BW children by an IQ gain from hybrid vigour. However, heterosis has too weak an effect to explain more than a bit of the heterogeneity [32].

The above discussion of Black adoptees’ IQs made one reviewer unhappy; they felt it was “selective” because “Flynn effect corrections are applied only to the East Asian groups, never to the Blacks”. However, there is a solid methodological reason for this: one *must* make Flynn effect corrections to interpret the three studies of East Asians, because those studies lacked comparison groups of adoptees of other races, forcing a comparison of the East Asian adoptees to the general population norm. At the same time, the studies with Black adoptees contained multiple groups which could be compared to *each other*, and such comparisons need no Flynn effect correction. The one possible exception is the MTRAS, afflicted by a hard-to-predict Flynn effect mentioned above. The published MTRAS reports do not have enough information to correct for that Flynn effect, so I take the published data as given while warning that a Flynn effect might have skewed them. This is better than the reviewer’s defective approach of taking sample-size-weighted racial averages of the means in my Table 1 (thereby double counting some of the data, because Weinberg et al.’s sample is a subset of Scarr and Weinberg’s) and indiscriminately subtracting 10 points from each average (neglecting the fact that the Flynn effect inflated IQs to different degrees in different samples).

## 6. Summarizing My Re-Analyses

I have now reviewed all of the transracial adoption studies cited by peer-reviewed papers as part of the race and IQ debate. Reviewing the data with a less selective eye, and making allowances for the Flynn effect, adoptive IQ boosts, and attrition in a longitudinal study, the results are equivocal. One study of East Asian adoptees documents an IQ advantage, while two more suggest IQ disadvantages. A sample-size-weighted average of the results would give a slight IQ disadvantage, though this result could be strengthened or reversed depending on what one assumes about the adoptive IQ boost in these samples. Meanwhile, one study of Black adoptees documents an IQ disadvantage, while two more suggest IQ advantages. Merging the studies’ results suggests an inconsistent IQ disadvantage, but that conclusion can only be a weak one since the studies are small, statistically heterogeneous, and open to alternative interpretations.

The data are too patchy and inconsistent for conclusive inferences, but taken together they are evidence against the hereditarian hypothesis. The net racial IQ differences are small and some disagree with the hereditarian model’s predictions. The nil hypothesis—that adoptees of different races have similar IQs when raised in the same environment—fits the data at least as well. For East Asian adoptees, my sample-size-weighted average IQ is about 97 (which I derive from midpoints of the IQ ranges in Table 2, subtracting 6 points for the adoptive boost), which would plausibly be a bit higher were malnutrition allowed for. Based on the studies of Black–Black and BW transracial adoptees I expect them to have mean IQs of about 92 and 100 respectively, but these estimates are shaky, undermined by heterogeneity and the Black adoptees in Scarr and Weinberg [13] having less salubrious rearing circumstances. The East Asian and Black averages could plausibly be within a couple of points of 100 if measured with more precision, and if no Black adoptees were raised in environments measurably unequal to those of White adoptees.

The studies reviewed so far have proven inconclusive. An obvious next step is to seek more data to see how well competing hypotheses accommodate them. The study list in van IJzendoorn et al.’s Table 1 [17] contains three more studies of the IQs of transracially adopted East Asians, namely Lien et al. [30], Wattier and Frydman [33], and Stams et al. [34]. My own searches turned up another study, Loman et al. [35], as well as Dalen et al. [36] and Lindblad et al. [37], two papers about entire cohorts of Swedish men. I now discuss the results of all six papers.

## 7. Lien et al., 1977

Lien et al. [30] essentially replicated Winick et al. [10], but studied Koreans adopted later. It confirmed a dose–response relationship between malnourishment and IQ deficits: 31 “severely malnourished” adoptees had a mean IQ of 95, 62 “moderately malnourished” adoptees a mean IQ of 101, and 39 “well-nourished” adoptees a mean IQ of 105 (pp. 1735–1736). Between 1973 and 1975 the children took the same IQ tests as those in Winick et al., but there is no precise breakdown of how many took each test. As such, the Flynn effect inflation of IQs could have been minute or could have been as high as 12 points. Subtracting a middling estimate of 6 points from these adoptees’ IQ averages implies an overall mean IQ of 95. Subtracting the effect of the adoptive IQ boost would shrink this average further, probably below 90.

## 8. Wattier and Frydman, 1985

Wattier and Frydman [33], like Frydman and Lynn [11], considered the IQ of a few Asian children adopted in Belgium. From the abstract: “the authors present a study made in 1983 on a sample of 28 children from South-East Asia and adopted four to twelve years earlier by Belgian francofone [sic?] families living in the province of Hainaut”. The rest of the paper is in French, making it hard for me to read it closely, but its summary table (on page 67 and unnumbered) is clear enough: the sample’s mean score on the WISC/WPPSI was 116.6 with a standard deviation of 11.04. The mean VIQ was 110.8 and the mean PIQ was 119.8. Making the same Flynn effect adjustment as for Frydman and Lynn [11] leads to a mean VIQ of 99 and a mean PIQ of 96–100, both less than the Belgian norm. Subtracting an adoptive boost effect would bring them lower.

## 9. Stams et al., 2000

The Stams et al. study [34] appears to be unique in having used one IQ test to compare East Asian transracial adoptees of both sexes to non-East Asian transracial adoptees. Specifically, in 1995 [38], 100 Sri Lankan adoptees, 11 Colombian adoptees and 36 South Korean adoptees, all raised by “Caucasian white” parents, took the abbreviated Revised Amsterdam Child Intelligence Test (RAKIT) at age 7, with their raw scores converted to standardized IQs “derived from a representative sample of 1415 children between age 4 and 11, drawn from the Dutch general school population in 1982” [34] (pp. 1026–1028). The children were all adopted reasonably close to birth, with the Sri Lankans having a mean adoptive age of 7 weeks (with an SD of 3 weeks) and the Koreans and Colombians a mean adoptive age of 15 weeks (SD 4 weeks). However, the Korean and Colombian adoptees “stayed in an institution or foster home after separation from their biological mother at birth, until their adoption”, while the Sri Lankan children “were in the care of their biological mother until their adoption” (p. 1026). Although “[l]ittle is known about the child-rearing conditions of the Sri Lankian [sic] infants”, “[a]necdotal evidence from parent reports” indicated that “pre- and post-natal care for the relinquishing mother and her baby were far from optimal […] and the health condition of the mother was deplorable in many cases” (p. 1027). This may explain the relatively low mean IQ of 104 for the Sri Lankan adoptees, compared to mean IQs of 112 and 115 for the Colombian and Korean adoptees respectively.

The adoptee–adoptee comparisons need no Flynn effect correction because the study’s adoptees were tested approximately simultaneously. However, a correction is necessary if one wishes to compare the adoptees to the RAKIT’s standardization sample. Wicherts et al. [39] (pp. 524–525) report that Dutch 5-year-olds gained 2.8 IQ points on the test from 1982 to “1992/1993”, though the 1992–1993 sample was marginally above average in SES; extrapolating that rate of gain across the 1982–1995 period, the Stams et al. adoptees’ IQs were inflated by 3–4 IQ points. This just about explains the elevated mean IQ of the Sri Lankan adoptees, but not the higher means of the Colombian and Korean adoptees.

## 10. Loman et al., 2009

Loman et al. [35] wished to observe how post-institutionalized adoptees from outside the US (“who were adopted at 12 months of age or older and spent 75% or more of their preadoptive lives in institutional care”) developed differently to “children internationally adopted early, predominantly from foster care” and non-adopted children “raised continuously in their biological families in the United States” (pp. 427–428). Their group of post-institutionalized adoptees was made up of 42 Russian and Eastern European (R&EE) adoptees, 41 Asian adoptees, seven South American adoptees, and a single Ethiopian adoptee.

As part of the project, the researchers extrapolated the children’s IQs from their performance on the WISC-III’s block design and vocabulary subtests, except for the children who “scored >1 SD below the mean on either WISC-III subtest”. The latter children then took the Leiter International Performance Scale-Revised, and their IQ was taken as their “Leiter Brief IQ score” instead (p. 429).

Table 3 of Loman et al. [10] broke down “Estimated IQ” by country of origin. Its mean and standard deviation were 99.6 ± 15.0 for the R&EE adoptees (*n* = 41), 95.6 ± 17.1 for the South American adoptees (*n* = 7), and 107.7 ± 18.3 for the Asian adoptees (*n* = 40). Loman et al.’s *F*-test of these gave a statistically insignificant result (“F(2,88) = 1.56, NS”), and their paper inferred “no region-of-origin differences in estimated IQ” (p. 431). However, a Welch’s *t*-test of the difference between the Asian and the R&EE adoptees (8.1 points with a standard error of 3.7 points) rejects the null hypothesis of zero difference (*p* = 0.033, although one can argue that the explicit significance level should be less than 0.05 to account for potential multiple comparisons).

This difference is meaningfully large and arguably statistically significant, but there is an obstacle to calling it a White-East Asian racial difference. The R&EE adoptees were presumably all White (coming from Russia, Romania, Bulgaria, Slovakia, Ukraine, Moldova, and Poland) but the Asian subsample comprised 22 Chinese children, three Filipino children, a Vietnamese child, and 15 Indian children, so it was roughly a 2:1 mixture of East Asian and South Asian adoptees. The observed difference is therefore one between White adoptees and an agglomeration of East Asian and South Asian adoptees.

I might also compare the adoptees to the general population, although the necessary Flynn effect adjustment is compromised by the administration of only two WISC-III subtests. The study’s IQ testing took place between 2005 and 2007 [40]. The WISC-III was standardized in 1989 [7] (p. 214) and the LIPS-R in 1995 and 1996 to match the 1993 US Census [41,42]. Therefore the WISC-III takers were supported by a Flynn effect of 4.8–5.4 points, and the LIPS-R takers by one of 2.7–3.6 points. Taking the midpoints of those two intervals, and weighting the midpoints using the fact that 23.1% of the post-institutionalized adoptees took the LIPS-R [35] (p. 429), the post-institutionalized adoptees would have had their average IQ estimates inflated by 4.6 points. Subtracting that figure from each subgroup mean gives estimated IQ means and standard errors of 95.0 ± 2.3 for the R&EE adoptees, 91.0 ± 6.5 for the South American adoptees, and 103.1 ± 2.9 for the Asian adoptees. Incorporating the expected adoptive boost, the Asian adoptees’ mean extrapolated IQ would probably be similar to that of the general population, and the mean extrapolated IQs of the R&EE and South American adoptees appreciably less.

## 11. Dalen et al., 2008, and Lindblad et al., 2009

Dalen et al. [36] and Lindblad et al. [37] are the only studies I have found which took a systematic national sample instead of relying on a convenience sample. I discovered the Lindblad et al. paper first but focus on Dalen et al.’s as its sample greatly overlaps Lindblad et al.’s and is over twice as large.

Dalen et al.’s “study population was drawn from all male residents in Sweden born between 1968 and 1976 who were conscripted before 20 years of age […] and were still residents in Sweden at follow-up in December 2001”, and had “complete information on all four intelligence test variables” (p. 1213). They divided the bounty of this expansive sampling frame into six groups: Korean adoptees, non-Korean foreign adoptees, domestic adoptees born in Sweden, non-adopted siblings of foreign adoptees, non-adopted siblings of domestic adoptees, and the general population, defined as “Swedish-born offspring of two Swedish-born parents with no record of ever having adopted a child”.

When registering for conscription, these men took “Enlistment battery 80”, “an intelligence test” which gave “a global score derived from the four subtests”; the global scores “had a Gaussian distribution of scores between 1 and 9” (p. 1214). Dalen et al.’s Table 2 gives the mean and standard deviation of the global scores within all six groups, which I transform to an IQ scale, taking the general population’s mean and standard deviation as 100 and 15 by definition. Table 3 summarizes the results.

Lindblad et al. mention (p. 303) that the non-Korean foreign adoptees were a mixture of Black (Ethiopian) adoptees, East Asian (Thai) adoptees, and adoptees who were neither Black nor East Asian nor White (Indian, Chilean, Sri Lankan, Colombian, and Ecuadorian adoptees, among others), so the non-Korean foreign adoptees are a suboptimal comparison group for the Korean adoptees. The domestic adoptees, however, were presumably almost all White, and they scored 6.2 points below the Korean adoptees. Selective placement likely plays a part in the latter result, as the domestic adoptees’ non-adopted siblings scored 7.5 points below the foreign adoptees’ non-adopted siblings.

I may also compare the Korean adoptees to the general population. (Unlike the older studies of East Asian adoptee IQ, the Flynn effect is irrelevant here because the adoptees were tested over the same period as the general population.) The Korean men scored only 1.5 IQ points above Swedish sons of non-adopting Swedish parents. This is rather less than the “10 or more points higher than [Korean and Vietnamese adoptees’] adoptive national norms” inferred by Rushton and Jensen [3] (p. 276) from older adoption studies. Indeed, although statistically significant because of the samples’ large size, the 1.5-point difference is scarcely practically significant, and would be reversed were one to allow for the usual adoptive IQ boost, especially a boost enhanced by selective placement.

## 12. Summarizing the Additional Studies

Lien et al. [30] and Wattier and Frydman [33] suggest a mean East Asian transracial adoptee IQ below 100. Stams et al. [34] is harder to interpret; although that study’s East Asian adoptees were tested alongside non-East Asian adoptees, the non-East Asian adoptees do not fit cleanly into the usual hereditarian racial framework of Whites, Blacks, and East Asians, so it’s unclear how to test the hereditarian hypothesis against these data. In any event, its 36 South Korean adoptees had a mean IQ 3 points higher than 11 Colombian adoptees, and 11 points higher than 100 Sri Lankan adoptees. These differences cannot be explained in terms of adoptive age, although the Sri Lankans may have suffered less healthy birth environments. Overall, the Colombian–Korean IQ difference remains marginal evidence in favour of the hereditarian hypothesis and against the nil hypothesis. Loman et al. [35] likewise hints at a higher IQ for Asian adoptees than for White and South American adoptees, but that finding is blurred by the Asian subsample including South Asians, the study’s use of incomplete test batteries, and the Flynn effect’s ability to explain most of the apparent Asian adoptee advantage. Dalen et al. [36] and Lindblad et al. [37], the largest studies of East Asian transracial adoptees’ IQs, found those IQs were only marginally higher than those of Swedish men in general, suggesting a mean IQ below 100 in the absence of the adoptive boost. Although Stams et al. [34] and Loman et al. [35] find that East Asian transracial adoptees outscored other adoptees, the overall drift of the additional studies is that East Asian transracial adoptees do not tend to have unusually good IQs in their adoptive nations. Appearances to the contrary are a result of ignoring their atypically salutary adoptive homes and of failing to accommodate the Flynn effect.

## 13. Conclusions

Drawing together this paper’s re-analyses, I conclude that East Asian adoptees raised by Western Whites score about on par with non-adopted Western Whites, and that there is no consistent IQ difference between Black adoptees raised by Whites and White adoptees raised by Whites. Meanwhile, some studies document East Asian adoptee samples with higher IQs than non-East Asian adoptee samples, but it is not clear that any offer a clean comparison of East Asian adoptees and White or Black adoptees in similar environments on complete IQ batteries.

These inferences must be provisional because the studies give conflicting results, most of the studies are small, and all are methodologically flawed. Only two of the papers reviewed here drew on samples of foreign adoptees that were nationally representative, and then only of males, and *then* only of the adoptees in the destination country, not of inhabitants of their birth country.

Indeed, given the obvious difficulties with ethically taking a random sample of newborns in one country and having them adopted into random foreign homes, it seems unlikely that any fully representative study will ever be done. It follows that transracial adoption studies are unlikely to conclusively settle the race and IQ debate, since commentators may always level the valid methodological objection of unrepresentative sampling. It is nonetheless worthwhile to correct misleading claims about transracial adoptee IQ data, which are still made: see Christainsen’s remark that “East Asians growing up in white households in the US and Belgium have tended to score considerably above the white mean in terms of intelligence” [43] (p. 168). (Christainsen wrote next that “East Asians, or at least the Chinese among them, also tend to be relatively quiescent *independently of their child-rearing environment* and also have less variable heart rates (Kagan, Resnick, [sic] and Snidman 1988)”. However, Kagan, Reznick and Snidman [44] (p. 167) studied “three cohorts of Caucasian children from working- and middle-class Boston homes”, not Chinese children.)

As well as correcting specific claims, my re-analyses enable a fresh comparison of the hereditarian model to the data. Provisionally, the hereditarian model fails to fit the data when one applies the level of standard applied by hereditarians such as Rushton, Jensen and Lynn. For instance, Rushton and Jensen [3] (p. 276) wrote that “[t]he culture-only model cannot explain” the “finding” that “Korean and Vietnamese children adopted into White homes, even though as babies many had been hospitalized for malnutrition, nonetheless grew to have IQs 10 or more points higher than their adoptive national norms”. That comparison was fallacious because it neglected the Flynn effect and the unrepresentative environments in which adoptees live. Allowing for both effects, I estimate that East Asian adoptees tend to have IQs about equal to the relevant norms, and possibly a little below. This is what a nil hypothesis, and presumably a “culture-only model”, would predict, but it violates the hereditarian expectation of superior East Asian IQ. Contrary to Rushton and Jensen’s [3] (p. 276) allegation that “support for the hereditarian model again comes from adding the East Asian data to the mix”, the hereditarian model has at least as much trouble with the East Asian data as with the Black data. The model is not definitively ruled out; the data are too weak for that. However, a hypothesis that fits these data, at least as well, is the nil hypothesis: adoptees of different races would have similar IQs if raised in the same environment. To the extent that the nil hypothesis is true, genes are not so likely to be the main cause of racial IQ differences.

## Figures and Tables

**Table 1:** Studies of IQs of transracial adoptees adopted by Whites: unadjusted results.

Study Group	*N*	Age at Testing	Reported IQ	
			Mean	Std. dev.
*Tizard, 1974*				
White	17	4.5	113.0	7.8
Black–White	7	4.5	119.9	16.6
*Winick, Meyer, and Harris, 1975*				
East Asian, “malnourished”	36	≥6	102	9.7 ^b^
East Asian, “moderately nourished”	38	≥6	106	12.5 ^b^
East Asian, “well-nourished”	37	≥6	112	9.0 ^b^
*Scarr and Weinberg, 1976*				
White	25	5–21	111.5	16.1
Black–White	68	4–16	109.0	11.5
Black–Black	29	4–16	96.8	12.8
*Clark and Hanisee, 1982*				
East Asian	25	2.5–6	120	16
*Moore, 1986*				
Black–White	14	7–10	116.5	8.2 ^c^
Black–Black	9	7–10	118.0	10.5 ^c^
*Frydman and Lynn, 1989*				
East Asian	19	6–14	118.7	10.4
*Weinberg, Scarr, and Waldman, 1992* ^a^				
White	16	15–31	105.6	14.9
Black–White	55	14–26	98.5	10.6
Black–Black	21	14–26	89.4	11.7

^a^ Weinberg, Scarr, and Waldman’s 1992 paper is a longitudinal follow-up to Scarr and Weinberg’s 1976 paper, so the former’s sample is a subset of that presented in the latter; ^b^ Standard deviations for Winick, Meyer, and Harris [10] read off graphically from a digital copy of their paper; my estimates are probably accurate to 0.4 IQ points; ^c^ Pooled standard deviations computed from separate standard deviations for male and female subgroups.

**Table 2:** Studies of IQs of transracial adoptees adopted by Whites: partially adjusted results.

Study Group	*N*	Mean IQ		Adjusted for
		Original	Adjusted	
*Tizard (1974)*				
White	17	113.0	–	nothing
Black–White	7	119.9	–	nothing
*Winick, Meyer, and Harris (1975)*				
East Asian, “malnourished”	36	102	91–102	Flynn effect
East Asian, “moderately nourished”	38	106	95–106	Flynn effect
East Asian, “well-nourished”	37	112	101–112	Flynn effect
*Scarr and Weinberg (1976)*				
White	25	111.5	–	nothing
Black–White	68	109.0	–	nothing
Black–Black	29	96.8	–	nothing
*Clark and Hanisee (1982)*				
East Asian	25	120	112–114	Flynn effect
*Moore (1986)*				
Black–White	14	116.5	–	nothing
Black–Black	9	118.0	–	nothing
*Frydman and Lynn (1989)*				
East Asian	19	110.6 (VIQ)	99 (VIQ)	Flynn effect
		123.5 (PIQ)	100–103 (PIQ)	
*Weinberg, Scarr, and Waldman (1992)*				
White	16	105.6	101.8	attrition
Black–White	55	98.5	98.3	attrition
Black–Black	21	89.4	90.1	attrition

**Table 3:** Cognitive-test scores of Swedish men, adopted and non-adopted.

Study Group	Dalen et al. (IQ)			Lindbland et al. (Stanine)	
	*N*	Mean	Std. dev.	*N*	Mean
General population	342,526	100	15	142,024	5.26
Korean adoptees	780	101.5	15.2	320	5.31
Non-Korean foreign adoptees	1558	88.6	13.7	1125	3.96
Foreign adoptees’ non-adopted siblings	357	108.5	14.2	190	6.17
Domestic adoptees	1153	95.3	15.1	not given	not given
Domestic adoptees’ non-adopted siblings	286	101.0	15.9	not given	not given

Results extracted from Table 2 of Lindblad et al.’s report [37] (which gives mean scores on an apparent stanine scale, but not standard deviations, preventing conversion of the means to an IQ scale) and computed from Table 2 of Dalen et al.’s report [36].
